# A Computed Tomographic Assessment of Osteitis of Sinus Bony Structures in Horses With Sinonasal Disorders

**DOI:** 10.3389/fvets.2020.00627

**Published:** 2020-09-11

**Authors:** Padraic M. Dixon, Coline Puidupin, Dewi Borkent, Tiziana Liuti, Richard J. M. Reardon

**Affiliations:** The Royal (Dick) School of Veterinary Studies and The Roslin Institute, The University of Edinburgh, Edinburgh, United Kingdom

**Keywords:** horse, equine sinus disease, sinonasal imaging, computed tomography, sinus bone inflammation, sinus osteitis

## Abstract

**Background:** Computed tomographic (CT) imaging has shown some horses with sinonasal diseases to have changes in their sinus bony structures. Scintigraphic and clinical evidence of sinus osteitis have also been reported. However, no study has objectively examined for the presence and degree of osteitis in equine sinonasal disease.

**Objectives:** To assess for the presence and extent of osteitis of sinus-related bony structures by examination of CT images of horses with clinically and sinoscopically confirmed unilateral sinonasal disease.

**Study Design:** Retrospective examination of CT images of horses with confirmed, mainly chronic (>2 month duration) unilateral sinus disease of different etiologies.

**Methods:** Bone thickness at designated sites of the maxillary bone (*n* = 3), frontal bone (*n* = 1), infraorbital canal (*n* = 2), and bony nasolacrimal duct (*n* = 1) were measured, as were the maximal diameters of the infraorbital canal and the bony nasolacrimal duct on both affected and control sides. Maxillary bone density (in Hounsfield Units) was also assessed bilaterally. Bone thickness was compared between affected and controlled sides using paired statistical tests.

**Results:** Bone was significantly thicker in the affected sinuses compared to the control sides at the three maxillary bone sites (all, *P* < 0.001) and at both infraorbital bone sites (both, *P* < 0.001), but not at the two most dorsal sites examined, i.e. frontal bone (*P* = 0.188) and bony nasolacrimal duct (*P* = −0.260) sites. Infraorbital canal and bony nasolacrimal duct diameters were significantly wider in the affected as compared to the control sides (*P* < 0.001 and *P* = 0.002, respectively). Maxillary bone density did not differ significantly between the affected (mean = 1,075 HU, SD = 230.01) and control (mean = 1,100, SD = 200.71) sides (*t*_(58)_ = −1.03, *P* = 0.306).

**Main Limitations:** Possible variation in selecting measurement sites. Variation in the severity and chronicity of sinonasal disease between horses.

**Conclusions:** Osteitis and enlargement of paranasal bony structures commonly occurs in horses with sinonasal disease and can explain the clinical presence of ipsilateral diffuse soft tissue facial swelling, epiphora, and scintigraphic evidence of bone inflammation in sinonasal disease.

## Introduction

Equine sinonasal disorders are often chronic (>2 months duration), usually unilateral disorders, with multiple etiologies that are non-responsive to conservative therapy (1–4). It is difficult to identify the underlying cause of the sinus disease in most cases by clinical examination and therefore diagnostic imaging including radiography (5), scintigraphy (6, 7), and computed tomography (CT) (8–15) are usually required for this purpose. Imaging is also used to identify which sinus compartments are affected and to identify intercurrent nasal disorders, including the presence of sino-nasal fistula, inspissated exudate, bone sequestrae, and infected nasal conchal bullae and hence these disorders could be more correctly termed sinonasal disorders (3, 4, 9, 12–14). Such imaging information allows appropriate treatment to be performed, which can include dental extraction and removal of exudate from the affected sinuses or nasal cavities.

An important early equine CT study, largely examining dental and alveolar changes in 18 cases of sinus disease by Henninger et al. (9), noted maxillary bone thickening and infraorbital canal distortion in most horses. A later CT study also noted maxillary bone osteitis in a horse with sinusitis (10). A recent large CT study showed changes in infraorbital canal mineralization, size, shape, and/or position in 78% of horses with sinus disorders (15).

**Figure 1:**
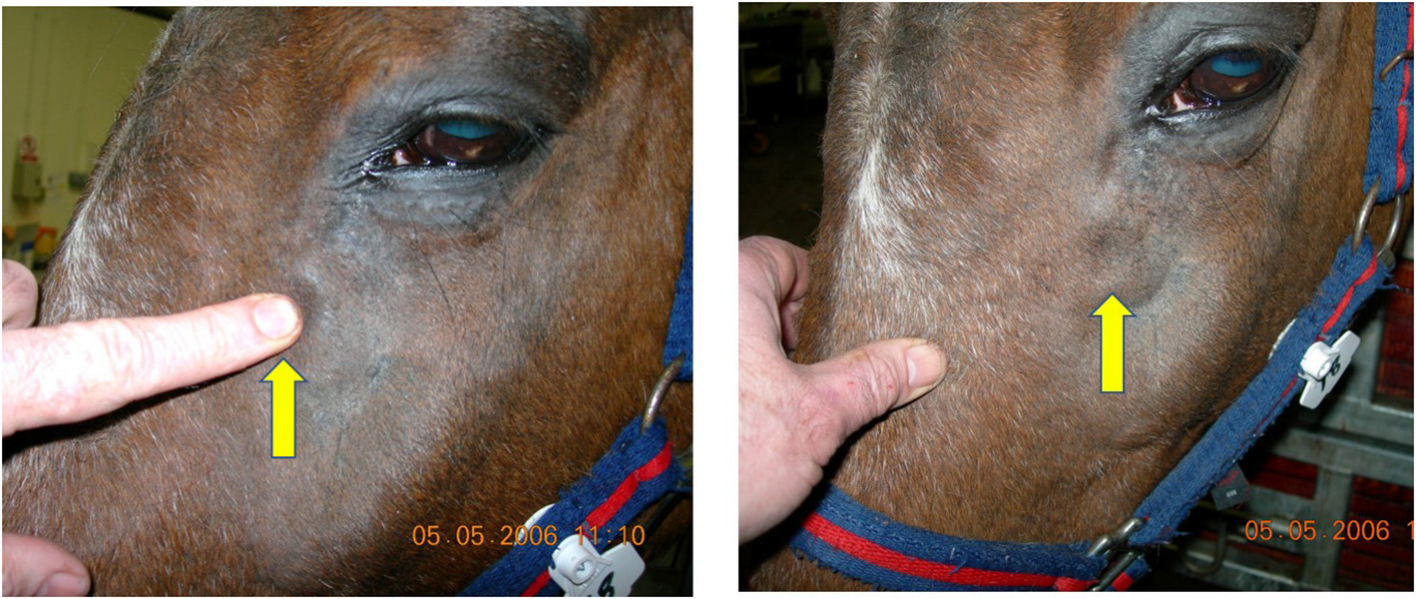
This horse with left-sided primary sinusitis has a soft tissue swelling over its left maxillary region that pits on digital pressure, indicating subcutaneous oedema. Maxillary bone sinusitis-related osteitis could explain such soft tissue facial swellings.

**Figure 2:**
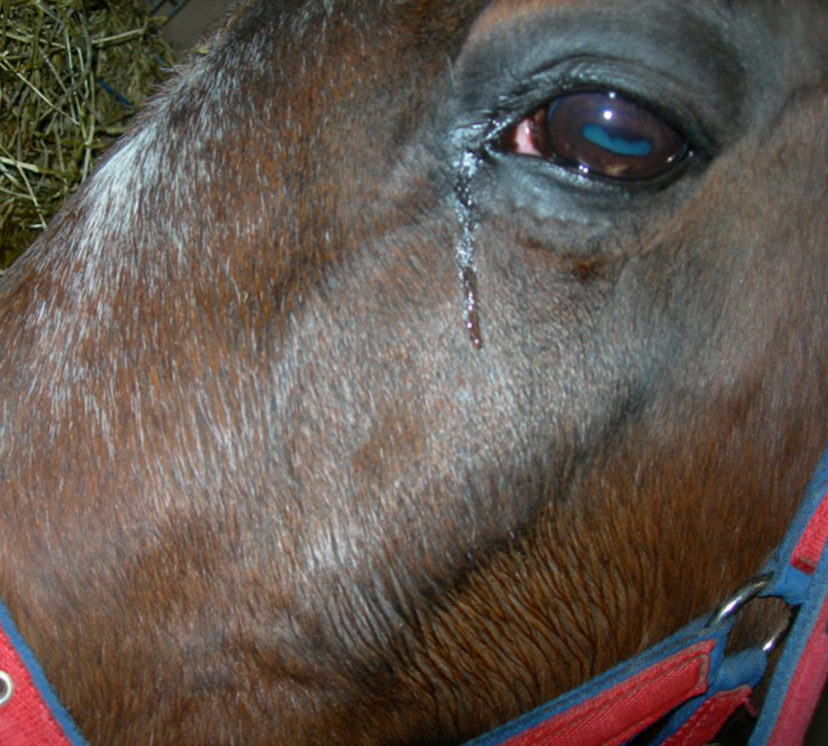
Horse with left sided sinusitis and ipsilateral epiphora. Such sinusitis-related epiphora were assumed to be caused by intra-sinus pressure from accumulated exudate, but may be caused by osteitis and partial obstruction of the nasolacrimal duct.

Scintigraphic imaging has shown that some horses with primary and dental sinusitis have unexplained, often focal areas of increased radioactive uptake (IRU), including of the maxillary bones, in addition to the alveolar bone changes in horses with dental sinusitis (7). Horses with the two most common types of sinus disease, i.e., primary and dental sinusitis do not have firm facial swellings that are often present in horses with sinus cysts, intra-sinus growths, and facial suture exostoses. However, some cases of primary and dental sinusitis develop unexplained low-grade, diffuse facial swellings (Figure 1), and others develop epiphora (Figure 2) (1, 2, 4). The latter has been assumed to be due to nasolacrimal duct compression from intra-sinus exudate accumulation.

**Figure 3:**
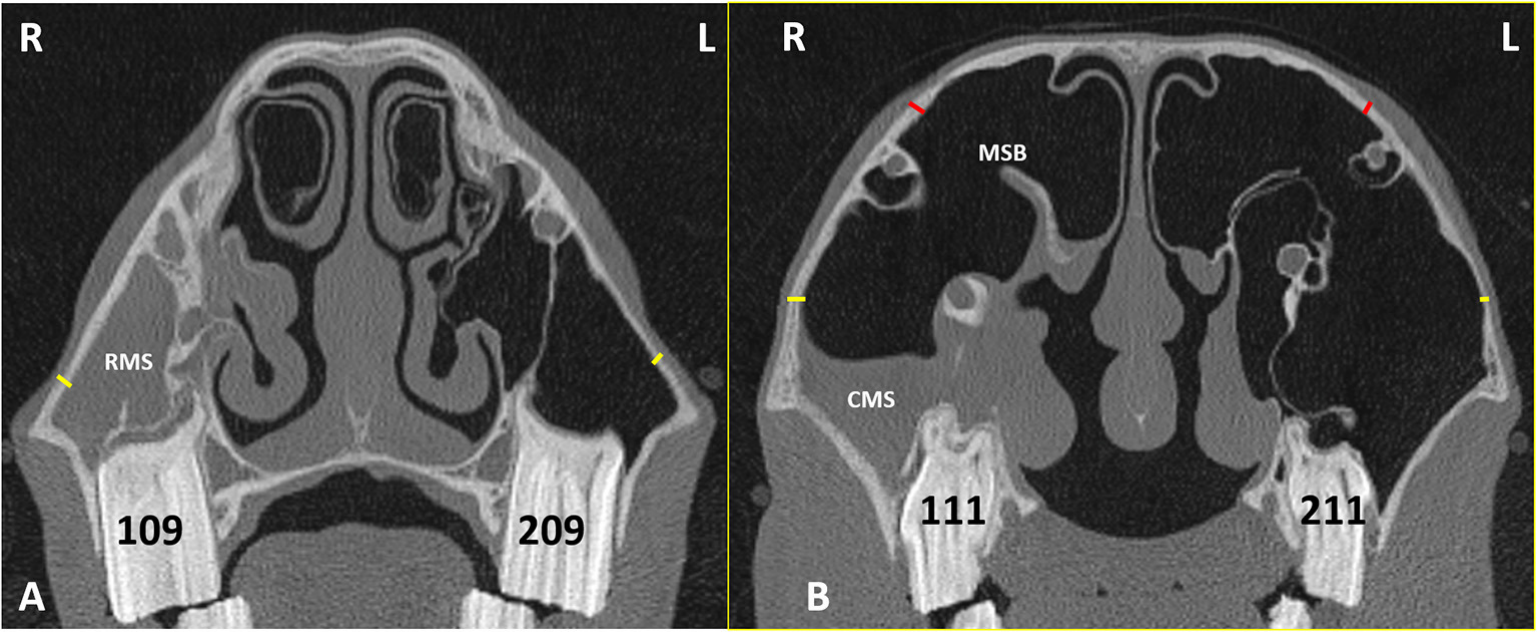
Transverse CT images of head of a horse with right-sided sinusitis obtained at the level of Triadan 09s **(A)** and Triadan 11s **(B)**. Hypoattenuating fluid is present within the right RMS and CMS. The right maxillary septal bulla (MSB) shows thickening of its bone and the overlying mucosa **(B)**. Maxillary bone measurement at the rostral maxillary sinus (RMB) **(A)** were obtained 2 cm dorsal to the facial crest on the affected right (R) and control left (L) sides (yellow lines). Maxillary bone measurement at the caudal maxillary sinus **(B)** were obtained 2 cm dorsal the maxillary crest (yellow lines) (CMBV) and also 1 cm dorsal the nasolacrimal canal (red lines) (CMBD) on the affected right (R) and control left (L) sides.

**Figure 4:**
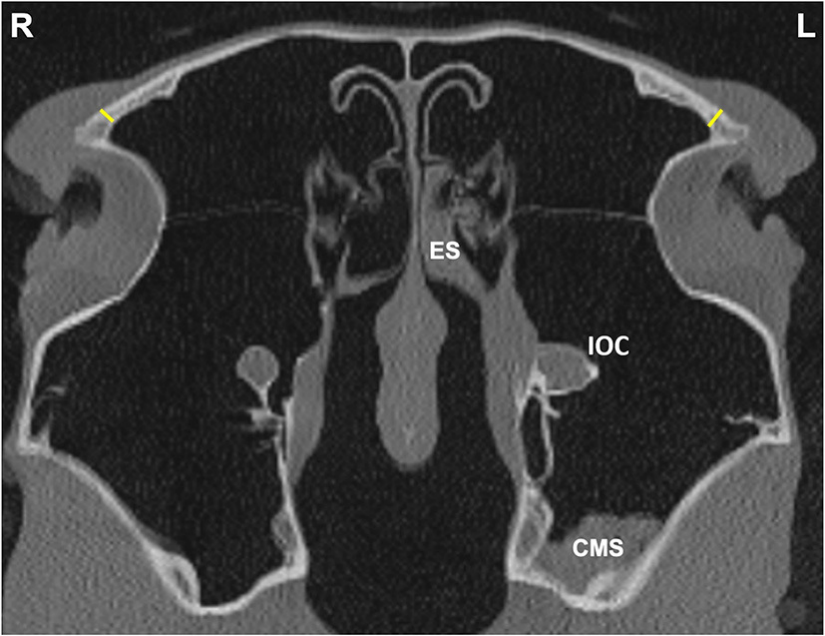
Transverse CT image of head of a horse with left-sided sinus disease, obtained at the level of the frontal sinus (FS). A small amount of hypoattenuating material is present within the left (L) caudal maxillary sinus (CMS) and ethmoidal sinus (ES) and there is a widened infraorbital canal (IOC). Frontal bone (FB) thickness was measured at a single site 1 cm dorsal to the junction between the medial aspect of the orbital rim and the caudal aspect of the nasal bone on the affected and contra-lateral sides (yellow lines).

Sinoscopy of horses with sinonasal disease sometimes shows thickening and calcification of the maxillary septal bulla (MSB) (formerly misnamed the *ventral conchal bulla*) (Figure 3B) and/or enlargement of the infraorbital canal (Figures 3B, 4). These two changes can cause difficulties or even prevent fenestration of the MSB, which is necessary to access the rostral maxillary sinus (RMS) and ventral conchal sinus (VCS), the compartments most commonly affected with sinus disorders (Authors personal observations).

The introduction of CT imaging for human chronic rhinosinusitis circa 30 years ago, allowed detailed assessment of sinus drainage pathways and of mucosal swelling not possible with radiography. It also clearly showed that in addition to mucosal inflammation, remodeling of the underlying bones occurred in many patients, that was histologically confirmed as a non-septic osteitis (16, 17). Since then, multiple studies have shown osteitis to occur with human chronic rhinosinusitis with a reported prevalence of 51% in all patients and of 76% in those that underwent sinus surgery, although the etiology and pathogenesis of this osteitis remain unclear (18–21). Many studies have examined the relationship between the degree of osteitis and the clinical severity and prognosis of human rhinosinusitis, especially in chronic refractory cases, and a number of sinus osteitis grading scales have been developed for this purpose (19–21). In addition to assessing bone thickness and the imaging appearance of sinus osteitis by CT, other authors have shown that an increase in bone density (H.U. values) in human sinusitis correlates with the degree histopathological bone changes present (22).

In contrast, no studies appear to have objectively assessed for the presence or extent of osteitis in equine sinonasal disease. The aim of this project was to retrospectively examine CT images of horses with unilateral sinonasal disease for evidence of osteitis of their maxillary and frontal bones, bony nasolacrimal duct and infraorbital canals by assessment of bone thickness, infraorbital canal, and bony nasolacrimal duct diameters, and of maxillary bone density, using the unaffected contra-lateral sinuses as controls.

## Materials and Methods

A retrospective examination was made of 60 randomly selected CT images of heads from horses of mean age 11.7 years (median age 11, range 5–26 years) with confirmed paranasal sinus disease (many also had intercurrent ipsilateral nasal disease) examined at the Equine Hospital of Edinburgh University Veterinary School between 2012 and 2018. The presence of sinus disease was confirmed by the presence of exudate draining from their sino-nasal ostia on standing nasal endoscopy in all cases; the sinoscopic detection of exudate in one or more sinus compartments in all cases and CT imaging (including the presence of fluid/soft tissue attenuation, i.e., from mucosal thickening and/or of accumulated exudate in some sinus compartments in every case).

The CT images were obtained under standing sedation using a Siemens Somaton Volume Zoom 4 slice or a Siemens Definition AS 64-slice (Siemens, Munich, Germany) in a helical scan mode using a 512 × 512 Matrix, 120 Kv, 300 mA, at a slice thickness of 1.5 mm. The images were re-examined by the authors using Horos® (Apple Corporation) software with the axes of the scans adjusted to obtain perpendicular transverse sections of the head for consistent measurements. Bone windows (H70) were used to review the images at a window width (WW) of 4,000 Hounsfield Unit (HU) and window level (WL) of 1,000 (HU).

### Bone Measurements

Linear bone measurements (using the mean of three measurements) were obtained perpendicular to the bone at the following sites.

#### Maxillary Bone Thickness at the RMS

Maxillary bone thickness of the RMS wall (**RMB**) was measured at a site level with the midpoint of maxillary Triadan 09 teeth, 2 cm above the dorsal aspect of the facial crest on the affected and contra-lateral sides (Figure 3A).

#### Maxillary Bone Thickness at the CMS (*N* = 2 Sites)

Maxillary bone thickness of the caudal maxillary sinus (CMS) wall was obtained at two sites; both level with the midpoint of maxillary Triadan 11s in the rostro-caudal plane on the affected and contra-lateral sides.

The ventral measurement (**CMBV**) was made 2 cm above the level of the maxillary crest.

The dorsal measurement (**CMBD**) was made 1 cm above the level of the nasolacrimal duct (Figure 3B).

#### Frontal Bone Thickness at the Frontal Sinus

Frontal bone (**FB**) thickness was measured at a single site 1 cm dorsal to the junction between the medial aspect of the orbital rim and the caudal aspect of the nasal bone on the affected and contra-lateral sides (Figure 4).

#### Infraorbital Canal

**Figure 5:**
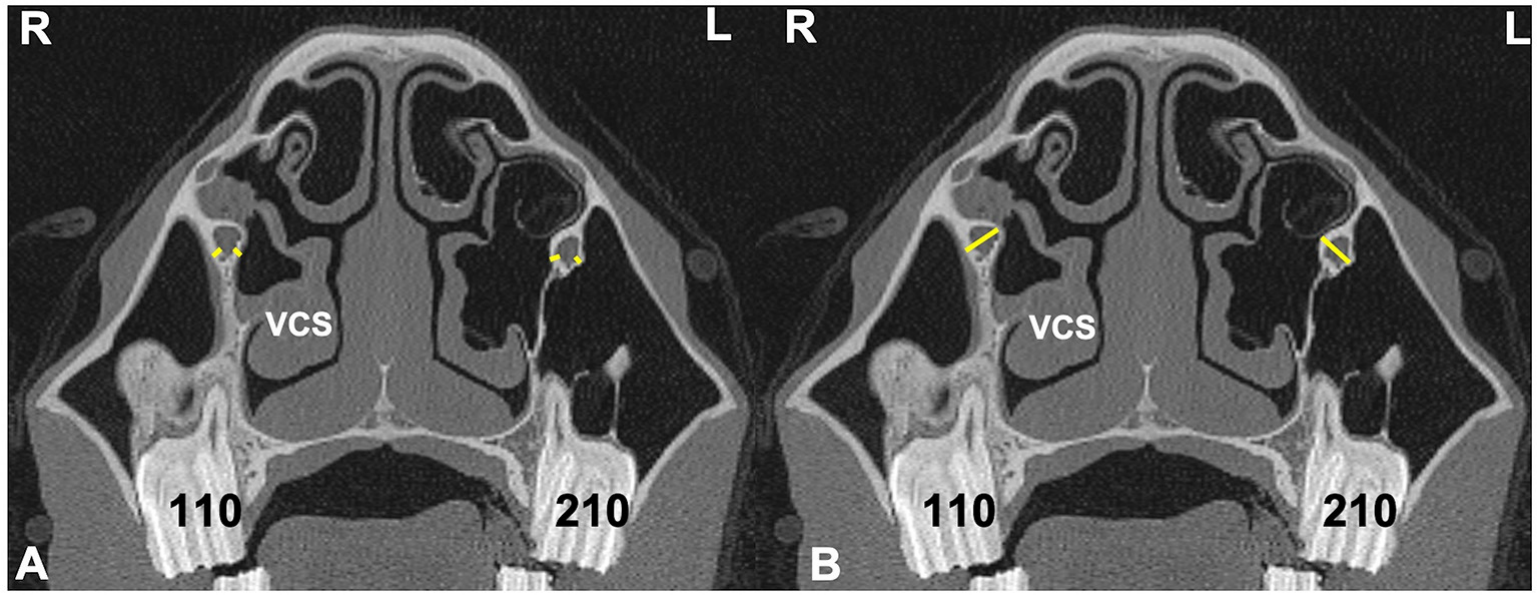
Transverse CT images of head of a horse with right-sided sinusitis obtained at the level of Triadan 10s. Some hypoattenuating material is present in the right ventral conchal sinus. **(A)** Sites of two measurements of infraorbital canal (IOC) wall thickness (IOCB 1 and 2) on the affected right (R) and control left (L) sides (yellow lines). **(B)** Sites of measurements of IOC diameter (IOCD) on the affected and control side (yellow lines).

The maximal infra-orbital canal (IOC) bony wall thickness within the sinuses was obtained at two sites (**IOCB1** and **IOCB2**) level with the midpoint of the Triadan 10s in the rostro-caudal plane, on the affected and contra-lateral sides (Figure 5A). The maximum IOC external diameter (**IOCD**) in the CMS was also measured bilaterally. (Figure 5B).

#### Bony Nasolacrimal Duct

**Figure 6:**
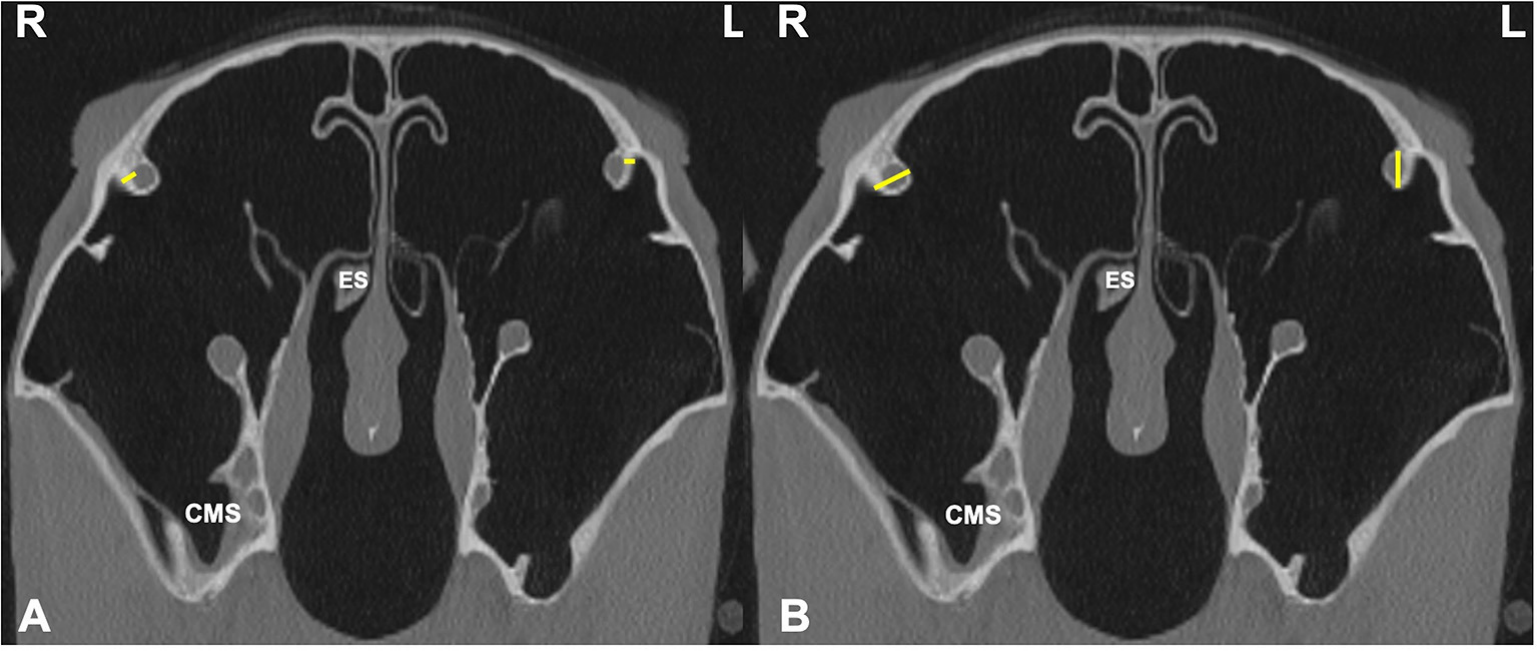
Transverse CT images of head of a horse with right-sided sinusitis, obtained at the level of the bony nasolacrimal ducts (NLD), caudal to the Triadan 11s. Small amounts of hypoattenuating material are present within the right caudal maxillary sinus and right ethmoid sinus (ES). **(A)** Measurements sites of the NLD wall bone thickness (NLDB) on the affected (R) and control (L) sides (yellow lines). **(B)** Measurements sites of NLD diameter (NLDD) on the affected (R) and control (L) sides (yellow lines).

The nasolacrimal duct (NLD) maximal bony wall thickness (**NLDB**) and its maximal external diameter (**NLDD**) were measured caudal to the Triadan 11s on the affected and control sides (Figures 6A,B).

**Figure 7:**
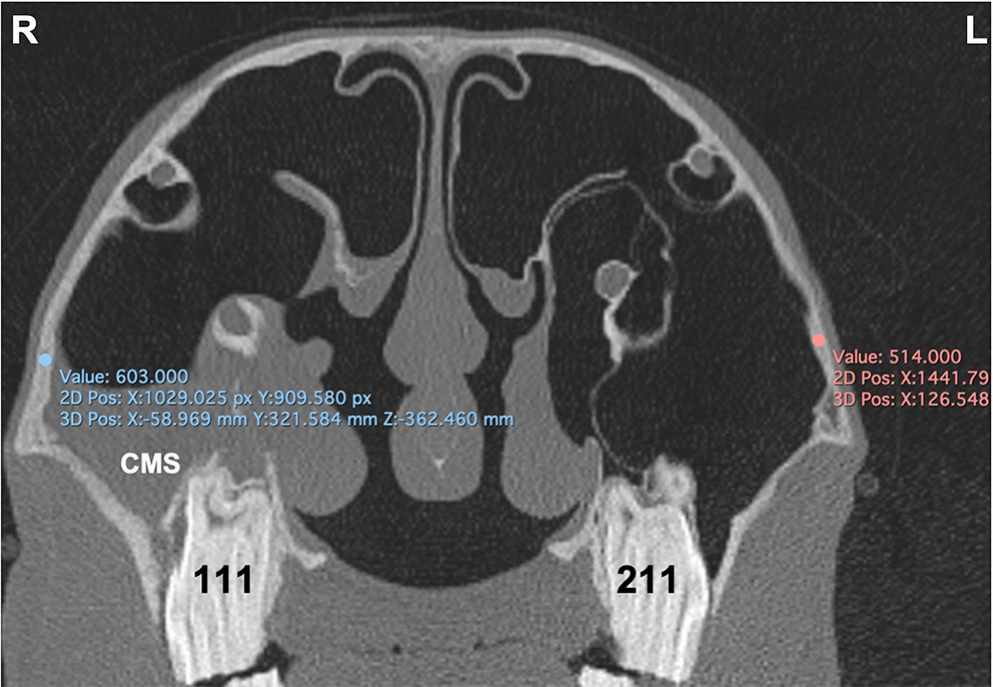
Transverse CT image of head of horse with right-sided sinusitis obtained at the level of mid aspect of Triadan 11s. Some hypoattenuating fluid is present within the right CMS. HU measurements of the maxillary bone at the caudal maxillary sinus were obtained 2 cm dorsal the maxillary crest on the affected right (R) and control left (L) sides.

**Figure 8:**
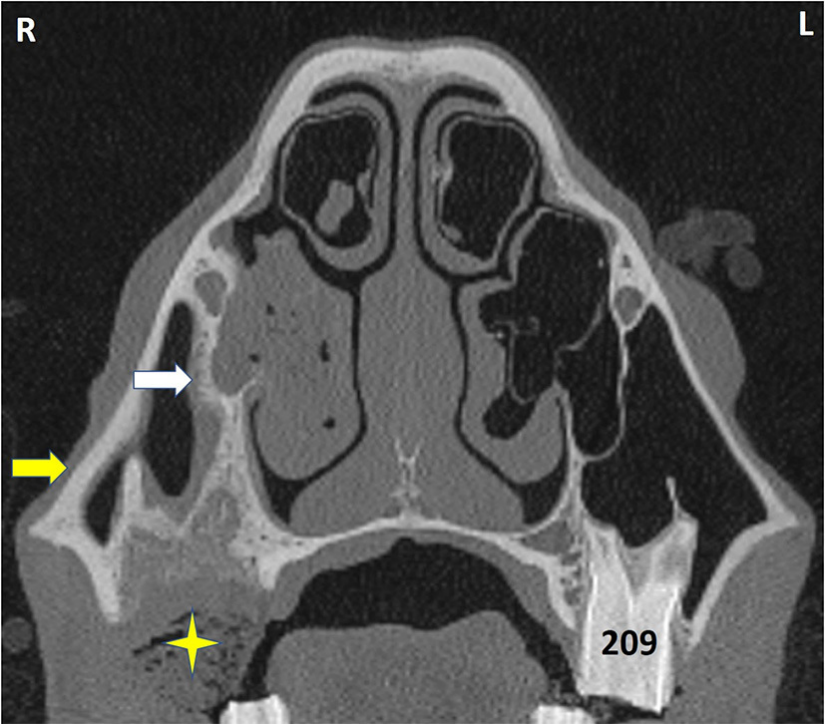
Transverse CT of a horse head at the level of Triadan 09. The horse had exodontia of 109 because of dental sinusitis 2 months previously but re-presented with an ipsilateral ventral nasal conchal bulla infection. The VCS contains hypoattenuating material with interspersed gas (shown to be inspissated exudate). The partly-healed post-extraction alveolus contains some food (star) and has grossly thickened and remodeled bone caused by the prior apical infection and exodontia. The bony septum connecting the alveolus and infraorbital canal (white arrow) is much thicker than on the contralateral side, as is its overlying mucosa. The maxillary bone (yellow arrow) is also grossly thickened. L, left; R, right.

Three measurements were made at each of the above sites, when there was evidence of sinus disease (e.g., mucosal thickening or accumulation of exudate) in adjacent sinus compartments. A preliminary examination of the 60 CTs showed involvement of the VCS and/or RMS in every case. However, other than the common presence of low volumes of hypoattenuating fluid in the dependent rostro-ventral aspects of the CMS and dorsal conchal sinuses (DCS) without any generalized mucosal swelling of these compartments (Figures 3, 4, 6–8) involvement (i.e., mucosal swelling and/or fluid filling) of the more dorsal caudal sinus compartments including DCS and frontal sinus (FS) (conchofrontal sinuses) was present in only 25/60 cases. Consequently, measurements of the more dorsally situated bony structures (frontal bone and the nasolacrimal duct) were not made in the 35 cases without inflammation of these areas, because including these measurements would give erroneous overall findings.

#### Bone Density

The density of the maxillary bone assessed in (HU) was measured at the caudal maxillary sinus 2 cm dorsal the maxillary crest at the level of midpoint of Triadan 11s on the affected and control sides (Figure 7).

### Repeated CT Examination

Two horses that had clinical resolution of their sinonasal disease had further head CTs performed more than 12 months later for unrelated head disorders and some of the above sinus measurements were repeated.

### Statistical Analyses

The means of the three measurements at each site were calculated. The spread of the mean values for each site subdivided by control and affected were analyzed graphically and using Shapiro–Wilks tests. Measures were compared between control and affected sides using paired *T*-Tests for normally distributed data and using Wilcoxon signed-rank tests. Significance was set as *P* < 0.05. All statistical analyses were performed in RStudio™.

## Results

### Cases

The CT images used in this study were obtained from cases of: dental sinusitis (*n* = 45), subacute primary sinusitis (*n* = 3), chronic (>2 months) primary sinusitis (*n* = 8), small intra-sinus cysts (*n* = 2), mycotic sinusitis (*n* = 1), and oromaxillary fistula (*n* = 1). Only cases with unilateral sinus disease were included to allow use of the contra-lateral sinuses for control measurements. Soft tissue facial swelling was present in 7/60 (11.7%) and epiphora in 4/60 (6.7%) cases.

**Figure 9:**
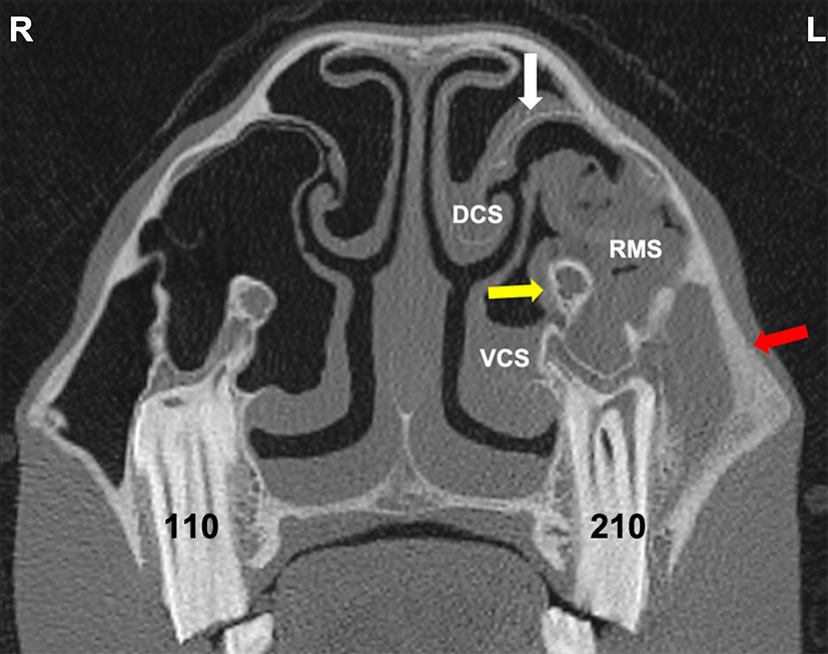
Transverse CT image through the rostral sinus compartments at the level of Triadan 10 of a horse with dental sinusitis caused by a Triadan 210 apical infection. The 210 has changes in its buccal root, widened periodontal space, and alveolar bone remodeling. There is resultant empyema of the VCS, DCS, and RMS (with gas bubbles in the latter). There is also thickening of the maxillary septal bulla bone and swollen mucosa on both sides (white arrow), some expansion and wall thickening of the infraorbital canal (yellow arrow) and grossly thickened maxillary bone (red arrow). L, left; R, right.

Many of these images showed grossly recognizable maxillary bone thickening and changes in infra-orbital canal and bony nasolacrimal duct appearance (Figures 8, 9).

Average measures and results of statistical comparisons between control and affected sides are shown in Table 1.

**Table 1:** Difference in measures of bone thickness and canal diameters between control and affected sides.

**Site**	**Sides compared**	**Control**		**Affected**		**Affected > control (%)**	**Wilcoxon signed rank *Z* statistic**	**Paired *T*-test *t*-statistic (df)**	***P*-value**
		**Median (mm)**	**Mean (SD) (mm)**	**Median (mm)**	**Mean (SD) (mm)**				
RMB	60	2.45		3.39		55 (91.67)	−6.27		**<0.001**
CMBV	25	2.61		3.05		20 (80.00)	−4.04		**<0.001**
CMBD	25	3.41		3.78		22 (88.00)	−4.28		**<0.001**
FB	19		3.00 (0.73)		3.20 (0.67)	11 (57.89)		−1.37 (18)	0.188
IOCB1	57	1.66		2.35		57 (82.46)	−5.39		**<0.001**
IOCB2	57	1.65		1.95		44 (77.19)	−3.92		**<0.001**
IOCD	60	12.07		14.20		55 (91.67)	−5.97		**<0.001**
NLDB	23	1.89		2.02		15 (65.22)	−1.13		0.260
NLDD	23		9.24 (2.24)		10.53 (2.39)	17 (73.91)		−3.50 (22)	**0.002**

*RMB, Maxillary bone at rostral maxillary sinus; CMBV, Maxillary bone at ventral site of caudal maxillary sinus; CMBD, Maxillary bone at dorsal site of caudal maxillary sinus; FB, frontal bone thickness; IOCB1, IOCB2, infraorbital canal bone thickness at two sites. IOCD, maximum IOC; NLDB, maximal nasolacrimal duct bone thickness; NLDD, maximum nasolacrimal duct diameter. Bold values indicate P values - standard term*.

**Figure 10:**
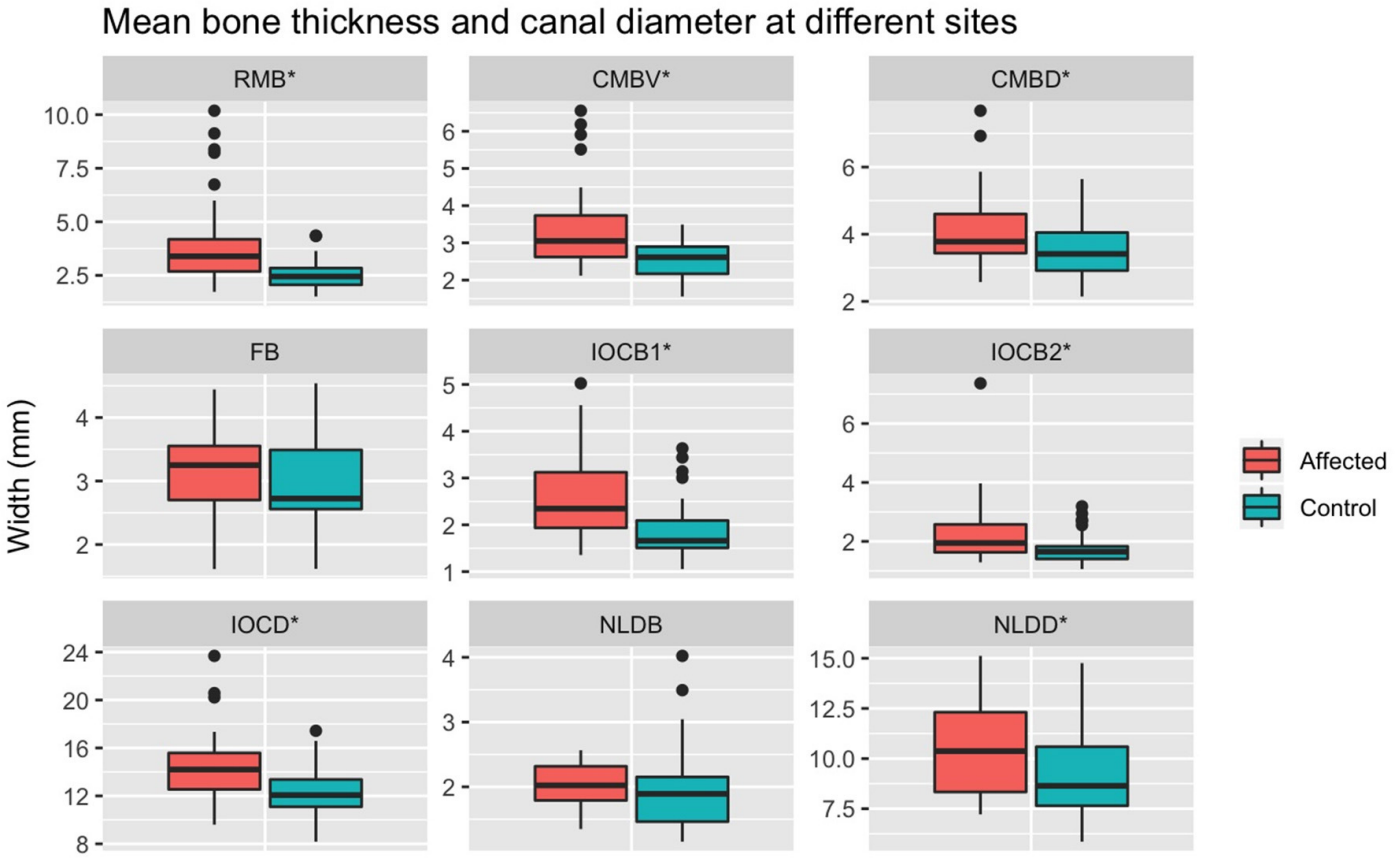
Mean measurements at different sites between control and affected sides. RMB, Maxillary bone at rostral maxillary sinus; CMBV, Maxillary bone at ventral site of caudal maxillary sinus; CMBD, Maxillary bone at dorsal site of caudal maxillary sinus; FB, frontal bone thickness; IOCB1, IOBC2, infraorbital canal bone thickness at two sites. IOCD, maximum IOC; NLDB, maximal nasolacrimal duct bone thickness; NLDD, maximum nasolacrimal duct diameter; *significant difference between control and affected sides.

The spread of bone thickness and canal diameter measures are shown in Figure 10. Bone was significantly thicker in affected than control sides at: RMB (*P* < 0.001), CMBV (*P* < 0.001), CMBD (*P* < 0.001), IOCB1 (*P* < 0.001), IOCB2 (*P* < 0.001), but not at FB (*P* = 0.188) or NLDB (*P* = −0.260). Canal diameters were significantly wider in affected than control sides for: IOCD (*P* < 0.001) and NLDD (*P* = 0.002; Table 1, Figure 10).

Bone density (HU) did not differ significantly between affected (mean = 1,075, SD = 230.01) and control (mean = 1,100, SD = 200.71) sides [*t*_(58)_ = −1.03, *P* = 0.306].

Two horses that had clinical resolution of their sinonasal disease had further head CTs performed over 12 months later for unrelated disorders. Bone measurements at four sites in the affected sinuses at the initial visits (mean thickness = 3.0 mm; range = 2.2–3.5 mm) were of reduced thickness (mean 1.9 mm; range = 1.5–1.9 mm) in normal appearing sinuses at their second visit.

## Discussion

This study has shown the presence of osteitis in diseased equine sinuses as manifested by significantly thicker maxillary and infraorbital canal bone thickness, and infraorbital canal and nasolacrimal ducts diameter as compared to the normal contralateral sides. The thickened bones had irregular new bone formation, with evidence of lysis in some areas. This osteitis appears inflammatory in nature with no CT or clinical evidence of bone infection. A CT study of 18 horses with sinus disease by Henninger et al. (9) recorded maxillary bone thickening, endosteal sclerosis, and irregular surfaces in most cases.

Two different CT scanners were used to obtain the images used in this study, including a 4 and a 64 slice unit. Although, the latter can produce higher quality images, this study used the same slice thickness and a bone window for both sets of images and so the results of both units are comparable for the purpose of this study.

The landmarks used for most bone measurement sites were individual cheek teeth. Because these teeth drift mesially (rostrally) with age (23), the measured bone sites, especially the maxillary bone sites would be more rostrally positioned in older horses. However, all maxillary bone measurement sites, both rostrally and caudally located, had very significantly (*p* < 0.001) increased bone thickness and so the possible age-related differences in bone measurement sites would not have affected the results of this study.

Horses with intra-sinus expansive lesions such as sino-nasal cysts or neoplasia usually have large facial swellings (1, 2, 4, 24). Cissell et al. (25) showed that horses with sinonasal neoplasia have osteolysis of adjacent cortical bone along with destructive changes of the nasal conchae, septum, and/or infraorbital canal (25). Unexplained, low-grade, diffuse facial swellings also occur in 24–45% horses with the two most common types of sinus disease, i.e., primary and dental sinusitis (1, 2) (Figure 1). The maxillary bone osteitis shown in horses with sinonasal disease in this study can explain these soft tissue facial swelling overlying the maxilla that were clinically present in 7/60 (11.7%) of the current cases. Maxillary osteitis also readily explains the recorded IRU of non-alveolar sinus bones in 60% of horses with primary sinusitis (7). In that study, this non-alveolar IRU lead to false-positive diagnoses of dental sinusitis by blinded observers in 40% of primary sinusitis cases, when focal areas of IRU overlay dental apices (7).

Unilateral epiphora was recorded in 13–34% of horses with primary or dental sinusitis respectively in two previous studies (1, 2). This reported epiphora may have been due to nasolacrimal duct osteitis rather than to pressure from intra-sinus exudate, because the sinuses as noted, are infrequently completely filled with exudate. Although the increased nasolacrimal duct bone thickness recorded in affected sides in this study was not-significantly different from the control sides, this may have been affected by the smaller number of cases in which this was measurable (23/60 cases had caudal sinus group involvement) and a larger study would be interesting.

Osteitis of the infraorbital canal wall and expansion of the canal diameter were also recorded within affected sinuses. However, there have been minimal clinical reports of trigeminal nerve-related signs such as headshaking or nasal rubbing in horses with sinus disease, that might be expected with such infraorbital canal involvement (1, 2, 9, 15). More recently, Edwards et al. (15) examined the head CTs of 218 horses of which 9% displayed idiopathic headshaking. They found bony infraorbital canal changes including: increased and decreased mineralization, deformity, displacement, and occasionally canal wall disruption in 86% of horses with adjacent disorders (mainly sinusitis). However, infra-orbital canal changes (including decreased mineralization in 29%) were also present in 37% of horses without adjacent disorders (15). The latter finding may indicate that some degree infraorbital canal demineralization is within a range of normality. In any event, these authors only found a weak association between the CT infraorbital canal changes and the presence of idiopathic headshaking.

Sinoscopic fenestration of the MSB can be impeded in some horses with sinonasal disease by the presence of an enlarged infraorbital canal (in addition to MSB sclerosis) (Authors personal observations). The current study confirmed the presence of increased bone thickness and increased diameter of the infraorbital canal in horses with sinus disease, in addition to the mucosal swelling also present on the infraorbital canal (Figures 3–5). The MSB bone is thin and irregular, and often barely detectable on radiographic or CT imaging in normal horses. Consequently, its thickness was not measured in this study. However, CT images of equine sinonasal disease show an osteitis-induced increase in MSB bone thickness in some horses, in addition to increased mucosal thickness on both sides (Figures 2, 3). Fenestrating a sclerotic MSB via sinoscopic portals can be difficult or even impossible if marked thickening and calcification of the MSB is present as a result of chronic osteitis, especially if an adjacent enlarged, distorted or displaced infraorbital canal further obstructs safe surgical access. If marked MSB sclerosis and infraorbital canal enlargement or displacement are apparent on CT imaging, surgeons may need to reconsider if that case needs treatment by sinusotomy rather than sinoscopically (authors personal observations).

Effective treatment of horses with sinonasal disease, such as dental extraction or removal of intra-sinus or intra-nasal inspissated exudate results in permanent clinical resolution of the disease in the vast majority of cases (26, 27). Presumably, the sinus-related osteitis also resolves. In two cases where repeat CT imaging was performed there was indication of osteitis resolution, but much larger, structured studies are needed to verify these observations. This apparent resolution of osteitis differs greatly from human chronic rhinosinusitis where many cases are refractory to treatment, with a strong correlation between the degree of sinus osteitis and number of surgical interventions, both features likely reflecting the presence of underlying non-responsive chronic disease, rather than being related to each other. These inflamed bones may even act as reservoirs of pro-inflammatory cytokines that perpetuate sinus inflammation (21).

Despite the increased maxillary bone thickness found in affected cases in this study, bone density did not differ between affected (mean = 1,075 HU) and control (mean = 1,100 HU) sides. This may reflect similar proportions of areas of increased and decreased mineralization in equine sinus osteitis, with both of these changes reported in the infraorbital canals of horses with sinusitis (15).

Histological studies in animal models of human sinus inflammation, (including in some models with induced bacterial sinusitis), and on surgically resected bone from human chronic rhinosinusitis patients show a non-septic sinus bone osteitis, with no evidence of bacterial bone invasion (16–18). Multiple other histological investigations have also shown a low-grade osteitis with new woven bone formation, fibrosis, inflammatory cells, and increased osteoblastic and osteoclastic activity that correspond to the imaging changes, including thickening and irregularity of the sinus walls (17–19, 22). In the absence of any evidence of direct bacterial involvement, this osteitis in human chronic rhinosinusitis patients appears to be stimulated by pro-inflammatory cytokines and other mediators released from the overlying inflamed sinus mucosa (19, 22).

The histology of osteitis in equine sinonasal disease does not appear to have been evaluated to date. However, portions of the MSB are always removed during its fenestration and these could be used in a future histological study on equine sinusitis-related osteitis.

## Conclusions

Osteitis with thickening of sinus related bones that appears inflammatory in nature was found in most cases of equine sinonasal disease and its presence may lead to soft tissue facial swelling, epiphora, and increased IRU of sinus bones. Recognition of nasolacrimal duct and MSB osteitis, enlargement or distortion is useful in planning the surgical treatment of some cases of sinonasal disease.

## Data Availability Statement

The raw data supporting the conclusions of this article will be made available by the authors, without undue reservation.

## Author Contributions

PD designed the study. CP, TL, and DB performed the study execution, RR performed the data analysis. All authors contributed to the data interpretation and manuscript preparation.

## Conflict of Interest

The authors declare that the research was conducted in the absence of any commercial or financial relationships that could be construed as a potential conflict of interest. The handling editor CS declared a past co-authorship with one of the authors PD.

## References

[1] Tremaine WH Dixon PM. Equine sinonasal disorders: a long term study of 277 cases. Part I - Historical, clinical and ancillary diagnostic findings. Equine Vet J. (2001) 33:274–82 10.2746/042516401776249615 11352350

[2] Dixon PM Parkin TD Collins N Hawkes C Townsend NB Fisher G. Historical and clinical features of 200 cases of equine sinus disease. Vet Rec. (2011) 169:439. 10.1136/vr.d4844 21868434

[3] Dixon PM Parkin TD Collins N Hawkes C Townsend N Tremaine WH. Equine paranasal sinus disease: a long-term study of 200 cases (1997-2009 Ancillary diagnostic findings and involvement of the various sinus compartments. Equine Vet J. (2012) 44:267–71. 10.1111/j.2042-3306.2011.00420.x 21812807

[4] O'Leary JM Dixon PM A review of equine paranasal sinusitis: aetiopathogenesis, clinical signs and ancillary diagnostic techniques. Equine Vet Educ. (2011) 23:148–59. 10.1111/j.2042-3292.2010.00176.x

[5] Gibbs C Lane JG Radiographic examination of the facial, nasal and paranasal sinus regions of the horse. II. Radiological findings. Equine Vet J. (1987) 33:49–58. 10.1111/j.2042-3306.1987.tb02648.x 3678193

[6] Weller R Livesey L Maierl J Nuss K Bowen IM Cauvin ER. Comparison of radiography and scintigraphy in the diagnosis of dental disorders in the horse. Equine Vet J. (2001) 33:49–58. 10.2746/042516401776767458 11191610

[7] Barakzai S Tremaine H Dixon PM. Use of scintigraphy for diagnosis of equine paranasal sinus disorders. Vet Surg. (2006) 35:94–101. 10.1111/j.1532-950X.2005.00118.x 16409416

[8] Tietje S Becker M Bockenhoff G. Computed tomographic evaluation of head diseases in the horse: 15 cases. Equine Vet J. (1996) 28:98–105. 10.1111/j.2042-3306.1996.tb01599.x 8706655

[9] Henninger W Frame M Willmann M Simhofer H Malleczek D Kneissl S. CT features of alveolitis and sinusitis in horses. Vet Radiol Ultrasound. (2003) 44:269–76. 10.1111/j.1740-8261.2003.tb00454.x 12816367

[10] Buhler M Furst A Lewis FI Kummer M Ohlerth S. Computed tomographic features of apical infection of equine maxillary cheek teeth: a retrospective study of 49 horses. Equine Vet J. (2014) 46:468–73. 10.1111/evj.12174 23991903

[11] Liuti T Smith S Dixon PM. Radiographic, computed tomographic, gross pathological and histological findings with suspected apical infection in 32 equine maxillary cheek teeth (2012-2015). Equine Vet J. (2017) 50:41–7. 10.1111/evj.12729 28772346

[12] Dixon PM Froydenlund T Luiti T Kane-Smyth J Horbal A Reardon RJM. Empyema of the nasal conchal bulla as a cause of chronic unilateral nasal discharge in the horse: 10 cases (2013-2014). Equine Vet. J. (2015) 47:445–49. 10.1111/evj.12322 25041424

[13] Liuti T Reardon R Smith S Dixon PM. An anatomical study of the dorsal and ventral nasal conchal bullae in normal horses: computed tomographic anatomical and morphometric findings. Equine Vet J. (2016) 48:749–55. 10.1111/evj.12516 26440763

[14] Kolos F Bodecek S Vyvial M Krisova S Mrackova M. Transnasal endoscopic treatment of equine sinus disease in 14 clinical cases. Equine Vet Educ. (2019). 10.1111/eve.1306828643340

[15] Edwards RA Hermans H Veraa S. Morphological variations of the infraorbital canal during CT has limited association with headshaking in horses. Vet Rad Ultrasound. (2019) 60:485–92. 10.1111/vru.12773 31161704

[16] Kennedy DW Senior BA Gannon FH Montone KT Hwang P Lanza DC. Histology and histomorphometry of ethmoid bone in chronic rhinosinusitis. Laryngoscope. (1998) 108:502–7. 10.1097/00005537-199804000-00008 9546260

[17] Giacchi RJ Lebowitz RA Yee HT Light JP Jacobs JB. Histopathologic evaluation of the ethmoid bone in chronic rhinosinusitis. Am J Rhinol. (2001) 15:193–97. 10.2500/105065801779954148 11453507

[18] Bhandarkar ND Sautter NB Kennedy DW Smith TL. Osteitis in chronic rhinosinusitis: a review of the literature. Int Forum Allergy Rhinol. (2012) 3:355–63. 10.1002/alr.21118 23258589

[19] Leung N Mawby TA Turner H Qureishi A. Osteitis and chronic rhinosinusitis: a review of the current literature. Eur Arch Otorhinolaryng. (2015) 273:2917–23. 10.1007/s00405-015-3817-0 26525884

[20] Snidvongs K McLachlan R Chin D Pratt E Sacks R Earls P. Osteitic bone: a surrogate marker of eosinophilia in chronic rhinosinusitis. Rhinology. (2012) 50:299–305 10.4193/Rhino12.022 22888488

[21] Snidvongs K Sacks R Harvey RJ. Osteitis in chronic rhinosinusitis. Curr Allergy Asthma Rep. (2019) 19:24. 10.1007/s11882-019-0855-5 30874957

[22] Dong Y Zhou B Huang Z Huang Q Cui S Li Y. Evaluating bone remodeling by measuring Hounsfield units in a rabbit model of rhinosinusitis: is it superior to measuring bone thickness? Int Forum Allergy Rhinol. (2018) 8:1342–8. 10.1002/alr.22205 30238647

[23] Liuti T Reardon R Dixon PM. Computed tomographic assessment of equine maxillary cheek teeth anatomical relationships, and paranasal sinus volumes. Vet Rec. (2017) 181:452. 10.1136/vr.104185 28893971

[24] Morgan RE Fiske-Jackson AR Hellige M Gerhauser I Wohlsein P Biggi M. Equine odontogenic tumors: Clinical presentation, CT findings, and outcome in 11 horses. Vet Radiol Ultrasound. (2019) 60:502–12. 10.1111/vru.12793 31359553

[25] Cissell DC Wisner ER Textor J Mohr FC Scrivani PV Theon AP. Computed tomographic appearance of equine sinonasal neoplasia. Vet Radiol Ultrasound. (2012) 53:245–51. 10.1111/j.1740-8261.2011.01913.x 22211373

[26] Tremaine WH Dixon PM. Equine sinonasal disorders: a long term study of 277 cases. Part 2: treatments and results of treatment. Equine Vet J. (2001) 33:283–89. 10.2746/042516401776249787 11352351

[27] Dixon PM Parkin TD Collins N Hawkes C Townsend N Tremaine WH. Equine paranasal sinus disease: a long-term study of 200 cases (1997-2009) treatments and long-term results of treatments. Equine Vet J. (2012) 44:272–76. 10.1111/j.2042-3306.2011.00427.x 21812808

